# P-503. Health Care Providers' Experiences with and Perceived Barriers to Implementation of Long-Acting Injectable Preexposure Prophylaxis

**DOI:** 10.1093/ofid/ofae631.702

**Published:** 2025-01-29

**Authors:** Elena Beideck, Ryan Gould, Douglas Krakower

**Affiliations:** Beth Israel Deaconess Medical Center, Brookline, Massachusetts; Beth Israel Deaconess Medical Center, Brookline, Massachusetts; Harvard Medical School, Boston, MA

## Abstract

**Background:**

Many patients eligible for preexposure prophylaxis (PrEP) for HIV are not yet prescribed it. Long-acting injectable cabotegravir (CAB-LA) may be a tool to increase PrEP coverage, though its uptake has been limited.

**Methods:**

We conducted an online national survey to evaluate providers’ experiences with prescribing and perceived barriers to implementing CAB-LA. The anonymous survey was administered from 07/2023 to 11/2023 to a convenience sample of providers affiliated with the AIDS Education and Training Centers. We used Fisher’s exact tests to assess for factors associated with availability and use of CAB-LA.

**Results:**

There were 89 total respondents from diverse professions (40% physicians, 60% non-physicians) from multiple regions (61% in the Northeast). Nearly half (46%) reported that at least 10% of their panel was uninsured and most (85%) reported that at least 40% of their panel had public insurance. Among providers, 57% reported their primary clinic site offered CAB-LA. Only 29% had patients on CAB-LA, and 10% had patients who tried CAB-LA and switched to oral PrEP. The most common perceived systems-level barriers to use of CAB-LA were concerns about insurance coverage (84%) and logistic constraints (78%). The most common perceived patient-related barriers were lack of patient requests (71%), concern for missed injections (68%), and cost (64%). The most common perceived reasons for patients to switch off of CAB-LA were burden of appointments (78%), injection site pain (67%) or other side effects (44%), and cost (33%). A majority (67%) reported that lack of training was at least a minor barrier to the prescribing of CAB-LA by providers at their practice site. Rates of CAB-LA availability and providers having prescribed CAB-LA did not vary by provider credentials, region, panel insurance mix, or rates of uninsured patients in providers’ panels (p-values all >0.05).

**Conclusion:**

This study highlights the modest rate of availability and the low rate of prescribing of CAB-LA. This suggests the need for improved strategies to implement CAB-LA, specifically surrounding cost, clinic logistics, patient awareness, and provider education. The rate of providers who have had patients switch off of CAB-LA suggests that patients may benefit from support to ease the burden of appointments and cost.

**Disclosures:**

**Douglas Krakower, MD**, FluidForm Bio: Stocks/Bonds (Private Company)|Gilead: Grant/Research Support|Matchbox Health: Stocks/Bonds (Private Company)|Medscape: Funding to develop educational content|Merck: Grant/Research Support

